# The Influence of HbA1c and Gestational Weight Gain on Pregnancy Outcomes in Pregnant Women With Gestational Diabetes Mellitus

**DOI:** 10.3389/fmed.2022.842428

**Published:** 2022-05-26

**Authors:** Qiuhong Zhang, Chee Shin Lee, Lixia Zhang, Qi Wu, Yunyan Chen, Danqing Chen, Lu Qi, Zhaoxia Liang

**Affiliations:** ^1^Obstetrical Department, Women's Hospital, School of Medicine, Zhejiang University, Hangzhou, China; ^2^Nanxun District People's Hospital, Huzhou, China; ^3^Huzhou Women and Children's Hospital, Huzhou, China; ^4^Department of Epidemiology, School of Public Health and Tropical Medicine, Tulane University, New Orleans, LA, United States

**Keywords:** gestational diabetes mellitus, glycated hemoglobin, gestational weight gain, pregnancy outcomes, HbA1c

## Abstract

**Background:**

To investigate the influence of HbA1c level and GWG on pregnancy outcomes in pregnant women with GDM.

**Methods:**

A total of 2,171 pregnant women with GDM were retrospectively included and categorized as follows: (1) normal (HbA1c <6%) and elevated (HbA1c ≥6%) HbA1c groups according to the HbA1c level in the second trimester, and (2) inadequate, appropriate, and excessive GWG groups according to the IOM guidelines.

**Results:**

In pregnant women with GDM, advanced age and high pre-pregnancy BMI were high-risk factors for elevated HbA1c. Pregnant women with elevated HbA1c had higher OGTT levels than those with normal HbA1c, and the risks of adverse pregnancy outcomes were higher (*P* < 0.05). The risks of primary cesarean section, hypertensive disorders during pregnancy, and macrosomia in pregnant women with excessive GWG were significantly higher than those with inadequate and appropriate GWG (*P* < 0.05). When GWG was appropriate, the risk of hypertensive disorders during pregnancy in the elevated HbA1c group was higher than that in the normal HbA1c group. When GWG was excessive, the risks of postpartum hemorrhage, macrosomia, and neonatal asphyxia in the elevated HbA1c group were significantly higher than in the normal HbA1c group (*P* < 0.05).

**Conclusion:**

Monitoring and controlling blood glucose levels have shown effectiveness in reducing the adverse pregnancy outcomes in women with GDM, particularly for those who had excessive GWG.

## Introduction

Gestational diabetes mellitus (GDM) refers to diabetes that appears or is diagnosed during pregnancy in women with normal glucose metabolism or potentially impaired glucose tolerance before pregnancy (1). With the improvement of living standards and the popularization of screening for gestational diabetes, the prevalence of GDM has been increasing over the years (2–4). It has become one of the main pregnancy complications that endanger the health of mothers and babies (5, 6). GDM not only increases the risk of adverse perinatal outcomes, such as macrosomia, intrauterine growth restriction, fetal malformation, neonatal respiratory distress syndrome, neonatal hypoglycemia, and so on, but also causes long-term complications in the offspring, including hypertension and type 2 diabetes (7–11).

Glycated hemoglobin (HbA1c) reflects average blood glucose levels during the preceding 8–12 weeks. As the gold standard for assessing long-term glycemic control, it has been widely used in blood glucose monitoring and management of people with diabetes. The increase in HbA1c levels is closely related to the adverse pregnancy outcome (12). In addition, the gestational weight gain (GWG) of pregnant women with GDM also has an important impact on the perinatal outcomes (13). In 2009, the American Institute of Medicine (IOM) formulated a reasonable range of GWG for pregnant women with different pre-pregnancy BMI values. It is indicated that the HbA1c level above 5% in the third trimester of pregnancy and excessive GWG are both risk factors for neonatal complications in women with gestational diabetes (14). However, in China, a few large-scale studies have focused on the effect of HbA1c on the outcomes in GDM, let alone prospective research about the effect of GWG on the pregnancy outcomes with different HbA1c levels.

This study retrospectively included 2,171 pregnant women with GDM, and aimed at exploring the effects of second-trimester HbA1c levels on pregnancy outcomes of GDM and analyzing the role of GWG on the pregnancy outcomes with different HbA1c levels, thus providing the basis for precise intervention and personalized, risk-stratified weight management for gestational diabetic women with different HbA1c levels.

## Materials and Methods

### Study Design and Data Sources

**Figure 1:**
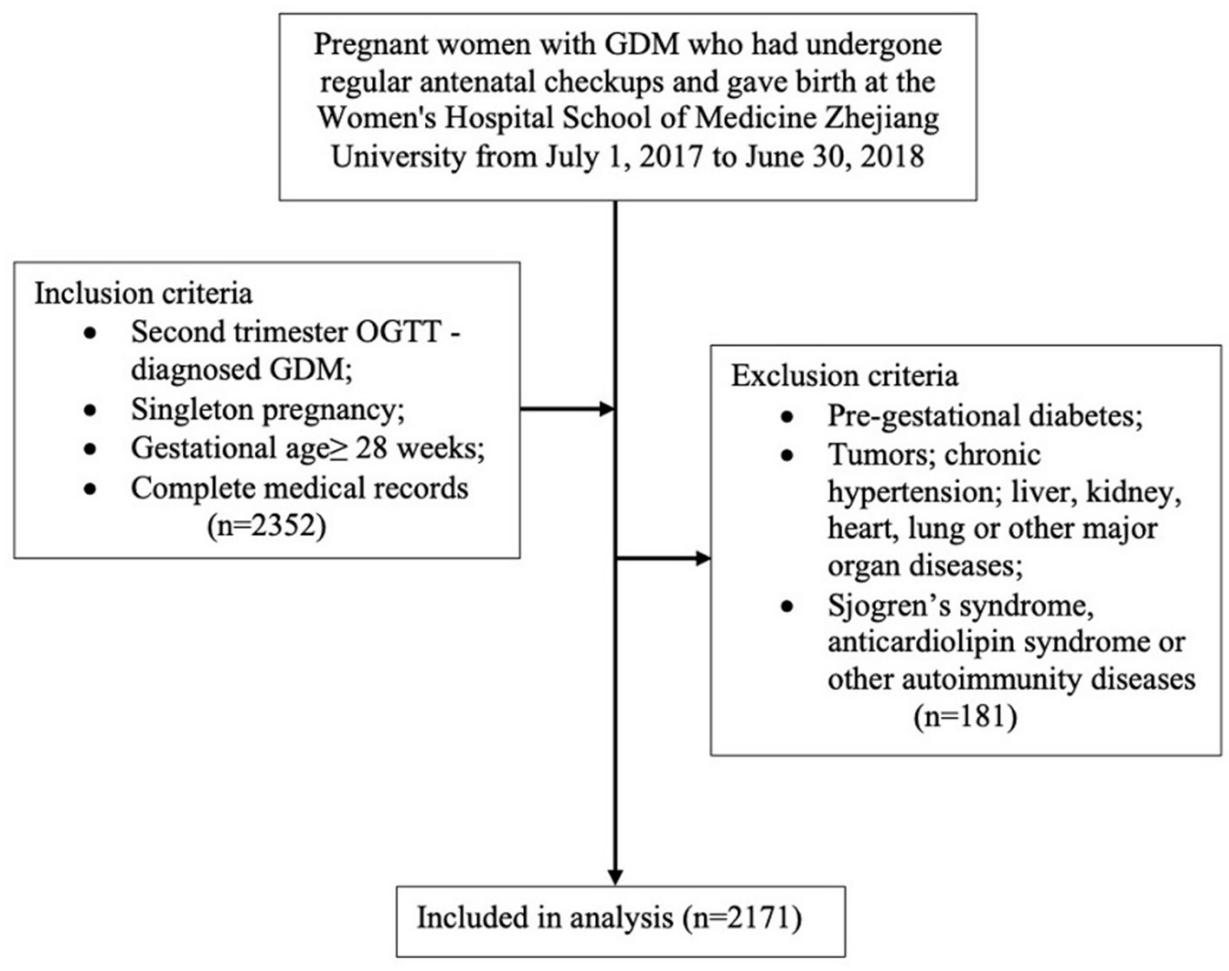
Participant flowchart.

A retrospective collection of pregnant women with GDM who had undergone regular antenatal checkups and gave birth at the Women's Hospital School of Medicine Zhejiang University from 1 July 2017 to 30 June 30 2018. Inclusion criteria were as follows: (1) diagnosis of GDM in the second trimester using the oral glucose tolerance test (OGTT); (2) singleton pregnancy; (3) gestational age at birth ≥28 weeks; and (4) complete medical records. Exclusion criteria were as follows: (1) coupled with pre-gestational diabetes; (2) coupled with chronic hypertension, or diseases of the liver, kidney, heart, lung, and other major organs, or coupled with tumors; (3) coupled with Sjogren's syndrome, anticardiolipin syndrome, or other autoimmune diseases. A total of 2,352 pregnant women met the inclusion criteria. After the application of the exclusion criteria, a total of 2,171 cases were eventually included in this study (Figure 1).

The relevant information regarding the pregnant women was retrieved through the electronic medical record system of the Women's Hospital School of Medicine, Zhejiang University, which included pregnant women's age, height, pre-pregnancy weight (within 1 month before pregnancy), gestational weight gain, pregnancy parity, OGTT value (fasting, 1 h, and 2 h after oral glucose load), second-trimester glycated hemoglobin level, mode of delivery, gestational age at birth, birth weight of newborn, and pregnancy complications, such as hypertensive disorders during pregnancy (including gestational hypertension, preeclampsia, and eclampsia), placental abruption, postpartum hemorrhage, intrahepatic cholestasis of pregnancy, neonatal asphyxia, etc.

The study was approved by the Human Ethics Committee at Women's Hospital, School of Medicine, Zhejiang University.

### Measurements and Calculations

The normal glucose values of fasting, 1-h and 2-h 75-g OGTT, according to IADPSG criteria, were <5.1 mmol/ L, 10.0 mmol/L, and 8.5 mmol/L, respectively (15). Any abnormal blood glucose level should be diagnosed as GDM. Body mass index (BMI) is defined as weight divided by the square of height (kg/m^2^). GWG (kg) is the difference between the weight before delivery and the weight before pregnancy. According to the 2009 IOM guidelines in the United States, the suggested GWG is 12.7–18.1 kg, 11.3–15.8 kg, 6.8–11.3 kg, and 5.0–9.1 kg for pregnant women with pre-pregnancy BMI categorized as underweight (BMI < 18.5 kg/m^2^), normal weight (BMI: 18.5 kg/m^2^−24.9 kg/m^2^), overweight (BMI: 25.0 kg/m^2^−29.9 kg/m^2^), and obese (BMI ≥ 30.0 kg/m^2^), respectively. One is considered to have inadequate GWG or excessive GWG if the GWG is less than or exceeding the appropriate value, respectively.

Gestational hypertension is the first incidence of high blood pressure at ≥20 weeks of gestation, with a systolic blood pressure of 140 mmHg and/or diastolic blood pressure of 90 mmHg in the absence of proteinuria or new signs of end-organ dysfunction. Preeclampsia is defined as a combination of one or more of the following conditions based on the diagnosis of gestational hypertension: proteinuria or other target organ dysfunction or uteroplacental function obstacle. Meanwhile, eclampsia is a seizure that occurs as a result of preeclampsia and cannot be explained by other factors. Placental abruption occurs when the placenta in its usual location becomes partly or totally separated from the uterine wall before the fetus is born after 20 weeks of pregnancy or during labor. Postpartum hemorrhage is defined as bleeding more than 500 ml after vaginal birth or more than 1,000 ml after cesarean delivery within 24 h of the birth of the fetus. Intrahepatic cholestasis of pregnancy is a pregnancy problem that manifests clinically as skin pruritus and increased bile acids in the second and third trimesters. The primary cesarean section is defined as the section that is performed on women who never had a cesarean delivery before. The neonatal Apgar score is presently used to diagnose newborn asphyxia, which is based on five indicators measured at 1 min after birth: heart rate, breathing, muscular tone, laryngeal reflex, and skin color. The normal score is 8–10 points, moderate asphyxia is given 4–7 points, and severe asphyxia is given 0–3 points. Macrosomia is defined as newborns with a birth weight ≥4,000 g.

### Statistical Analysis

**Figure 2:**
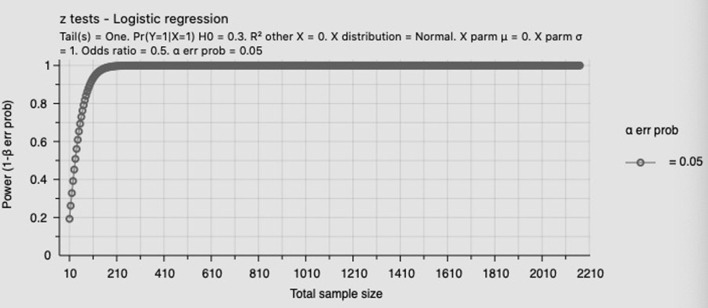
Power analysis for total population.

**Figure 3:**
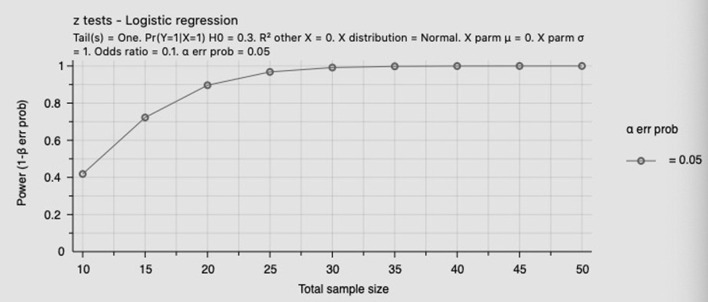
Power analysis for high HbA1c group.

The data of the normal distribution were expressed as x ± s, and the difference between the means of the two groups was analyzed by the *t*-test. Count data were expressed in frequency and rate, and the difference between groups was compared using the chi-squared (χ^2^) test. Multivariate logistic regression analysis was performed to evaluate the effect of glycated hemoglobin and GWG on pregnancy outcomes in pregnant women with GDM, while Fisher's exact test was used to examine the effect of GWG on the pregnancy outcomes of groups with different levels of HbA1c. A *p*-value of 0.05 or less was considered to be statistically significant. All statistical analyses were done using SPSS Statistics 25.0. Power analysis for total population and high HbA1c group was shown in Figures 2, 3.

## Results

### Comparison of General Clinical Characteristics and Pregnancy Outcomes in Two Groups With Different Glycated Hemoglobin Levels

We analyzed the risks factors of adverse pregnancy outcomes between groups with normal and elevated HbA1c levels. The results showed that the age, pre-pregnancy BMI, OGTT blood glucose level, primary cesarean section, and newborn's birth weight were significantly higher in the pregnant women of elevated HbA1c group when compared to those of the normal group, while the gestational age at birth was found to be decreased (*P* = 0.031, *P* = 0.000, *P* = 0.000, *P* = 0.016, *P* = 0.004, respectively). Meanwhile, the risk of hypertensive disorders during pregnancy, postpartum hemorrhage, placental abruption, neonatal asphyxia, and macrosomia was also significantly higher in the elevated HbA1c group (*P* = 0.007, *P* = 0.018, *P* = 0.044, *P* = 0.000, *P* = 0.004) (Table 1).

**Table 1:** Comparison of general clinical characteristics and pregnancy outcomes between two groups with different glycated hemoglobin levels.

		**Normal HbA1c** ***N*** **= 2122**	**Elevated HbA1c** ***N*** **= 49**	***P***
Age (Years)		32.38 ± 4.44	33.78 ± 5.24	0.031 ^*^
Gravidity		2.29 ± 1.28	2.43 ± 1.32	0.469
Parity		0.52 ± 0.53	0.55 ± 0.68	0.665
Pre-pregnancy BMI (kg/m^2^)		21.73 ± 3.12	24.67 ± 4.40	0.000^*^
Gestational weight gain (kg)		12.50 ± 4.12	12.64 ± 5.02	0.469
OGTT blood glucose level (mmol/L)	Fasting	4.66 ± 0.48	5.35 ± 0.68	0.000^*^
	1 h	9.97 ± 61.28	11.11 ± 1.42	0.000^*^
	2 h	8.67 ± 1.16	9.23 ± 1.08	0.001^*^
Gestational age at birth (weeks)		38.35 ± 1.60	37.69 ± 1.77	0.005^*^
Birth weight (g)		3292.04 ± 497.18	3500.00 ± 584.69	0.004^*^
Methods of delivery	Vaginal delivery	977 (46.0 %)	15 (30.6 %)	0.100
	Forceps delivery	70 (3.3 %)	2 (4.1 %)	
	Cesarean section	1,075 (50.7 %)	32 (65.3 %)	
Primary cesarean section		578 (27.2 %)	21 (42.9 %)	0.016^*^
Intrahepatic cholestasis of pregnancy		97 (4.6 %)	1 (2.0 %)	0.399
Hypertensive disorders during pregnancy		164 (7.7 %)	9 (18.4 %)	0.007^*^
Postpartum hemorrhage		78 (3.7 %)	5 (10.2 %)	0.018^*^
Placental abruption		22 (1.0 %)	2 (4.1 %)	0.044^*^
Neonatal asphyxia		19 (0.9 %)	4 (8.2 %)	0.000^*^
Macrosomia		129 (6.1 %)	8 (16.3 %)	0.004^*^

* *P < 0.05*.

### Comparison of Pregnancy Outcomes in Gestational Diabetic Women With Different Gestational Weight Gain During Pregnancy

By comparing the incidence of pregnancy outcomes among the three groups with different gestational weight gain, we found that the risks of hypertensive disorders during pregnancy, macrosomia, and primary cesarean section were significantly higher in the GDM women of the excessive GWG group when compared to those of inadequate and appropriate GWG groups (*P* = 0.000) (Table 2).

**Table 2:** Comparison of pregnancy outcomes in gestational diabetic women with different weight gains during pregnancy.

		**Inadequate GWG** ***N*** **= 815**	**Appropriate GWG** ***N*** **= 897**	**Excessive GWG** ***N*** **= 459**	***P***
Methods of delivery	Vaginal delivery	394 (48.3 %)	418 (46.6 %)	180 (39.2 %)	0.022
	Forceps delivery	29 (3.6 %)	29 (3.2 %)	14 (3.1 %)	
	Cesarean section	392 (48.1 %)	450 (50.2 %)	265 (57.7 %)	
Primary cesarean section		198 (24.3 %)	239 (26.6 %)	162 (35.3 %)	0.000^*^
Intrahepatic cholestasis of pregnancy		44 (5.4 %)	41 (4.6 %)	13 (2.8 %)	0.105
Hypertensive disorders during pregnancy		43 (5.3 %)	67 (7.5 %)	63 (13.7 %)	0.000^*^
Postpartum hemorrhage		24 (2.9 %)	36 (4.0 %)	23 (5.0 %)	0.169
Placental abruption		8 (1.0 %)	13 (1.4 %)	3 (0.7 %)	0.379
Neonatal asphyxia		6 (0.7 %)	10 (1.1 %)	7 (1.5 %)	0.409
Macrosomia		28 (3.4 %)	56 (6.2 %)	53 (11.5 %)	0.000^*^

* *P < 0.05*.

### The Influence of HbA1c and GWG on Pregnancy Outcomes in GDM Women

Through multivariate logistic regression analysis, we found that high HbA1c levels increased the risk of primary cesarean section, hypertensive disorders during pregnancy, and macrosomia. GDM women with more GWG have been shown to be at increased risk of primary cesarean section, intrahepatic cholestasis of pregnancy, and macrosomia. The differences were found to be statistically significant (*P* < 0.05) (Table 3).

**Table 3:** Influence of glycated hemoglobin and gestational weight gain on pregnancy outcome of pregnant women with GDM.

	**β value**	**OR**	**95%CI**	***P*** **-value**
**Primary cesarean section**				
HbA1c	0.440	1.552	(1.174, 2.052)	0.002^2^*^^
GWG	0.035	1.035	(1.012, 1.059)	0.002^*^
**Intrahepatic cholestasis** **of pregnancy**				
HbA1c	−0.022	0.942	(0.537, 1.782)	0.942
GWG	−0.059	0.943	(0.895, 0.993)	0.025^*^
**Hypertensive disorders** **during pregnancy**				
HbA1c	1.290	3.634	(2.357, 5.603)	0.000^*^
GWG	0.033	1.034	(0.997, 1.072)	0.070
**Postpartum hemorrhage**				
HbA1c	0.357	1.428	(0.756, 2.698)	0.272
GWG	0.048	1.050	(0.999, 1.103)	0.057
**Placental abruption**				
HbA1c	0.914	2.494	(0.842, 7.385)	0.099
GWG	−0.023	0.978	(0.886, 1.078)	0.651
**Neonatal asphyxia**				
HbA1c	0.602	1.825	(0.575, 5.792)	0.307
GWG	0.083	1.087	(0.996, 1.186)	0.062
**Macrosomia**				
HbA1c	1.241	3.460	(2.138, 5.598)	0.000^*^
GWG	0.089	1.093	(1.052, 1.136)	0.000^*^

* *P < 0.05*.

*OR values were adjusted for maternal age, gravidity, parity, and pre-pregnancy BMI*.

### The Effect of Gestational Weight Gain on Pregnancy Outcomes in Different Groups of HbA1c

When pregnant women with GDM had inadequate GWG, there was no significant difference in the risk of hypertensive disorders during pregnancy, postpartum hemorrhage, placental abruption, macrosomia, and neonatal asphyxia between the normal and elevated HbA1c groups. However, the risk of hypertensive disorders during pregnancy in the elevated HbA1c subgroup was significantly higher in the appropriate GWG group (*P* = 0.032). In addition, in the excessive GWG group, the incidence of postpartum hemorrhage, neonatal asphyxia, and macrosomia was also significantly higher in the elevated HbA1c subgroup than in the normal HbA1c subgroup (*P* = 0.009, *P* = 0.044, *P* = 0.027, *P* = 0.003) (Table 4).

**Table 4:** Effect of gestational weight gain on the pregnancy outcomes of different groups based on HbA1c levels.

	**Hypertensive disorders during pregnancy**	**Post-partum hemorrhage**	**Placental abruption**	**Primary cesarean section**	**Neonatal asphyxia**	**Macrosomia**
**Inadequate GWG (*****n*** **= 815)**						
Normal HbA1c	41 (5.1 %)	23 (2.9 %)	7 (0.9 %)	191 (23.8%)	5 (0.6 %)	28 (3.5 %)
Elevated HbA1c	2 (14.3 %)	1 (7.1 %)	1 (7.1 %)	7 (50.0%)	1 (7.1 %)	0
P	0.166	0.344	0.130	0.051	0.099	/
**Appropriate GWG (*****n*** **= 897)**						
Normal HbA1c	63 (7.2 %)	30 (4.1 %)	12 (1.4 %)	232 (26.4%)	9 (1.0 %)	54 (6.1 %)
Elevated HbA1c	4 (23.5 %)	0	1 (5.9 %)	7 (41.2%)	1 (5.93 %)	2 (11.8 %)
P	0.032	/	0.222	0.174	0.175	0.287
**Excessive GWG (*****n*** **= 459)**						
Normal HbA1c	60 (13.6 %)	19 (4.3 %)	3 (0.7 %)	155 (35.1%)	5 (1.1 %)	47 (10.7 %)
Elevated HbA1c	3 (16.7 %)	4 (22.2 %)	0	7 (38.9%)	2 (11.1 %)	6 (33.3 %)
*P*	0.724	0.009^*^	/	0.803	0.027^*^	0.003^*^

* *P < 0.05*.

## Discussion

The second-trimester HbA1c value ≥6% and gestational weight gain exceeding the IOM recommendations were risk factors for adverse pregnancy outcomes in pregnant women with gestational diabetes. In this study, we noted a significantly increased risk of adverse pregnancy outcomes among pregnant women with GDM in the elevated HbA1c group and excessive GWG group compared to those in the normal HbA1c group and appropriate and inadequate GWG groups, respectively. Moreover, GDM women with both high HbA1c levels and excessive GWG had a higher risk of postpartum hemorrhage, neonatal asphyxia, and macrosomia.

Gestational diabetes is one of the most common complications of pregnancy (5). In addition to the short-term risks during pregnancy and childbirth, the risk of long-term adverse outcomes in women with gestational diabetes and their offspring, such as diabetes, obesity, cardiovascular and metabolic diseases, disorders of blood glucose regulation in children and adolescents, and other metabolic syndromes, also increased significantly (10, 11, 16, 17). Therefore, targeted intervention strategies and measures to prevent the occurrence and development of GDM in high-risk groups, along with further management and control of hyperglycemia and weight during pregnancy, are vital to improving the glucose metabolism of women and their offspring, thus reducing the risk of cardiovascular and metabolic diseases.

Although OGTT is the gold standard for the effective diagnosis of gestational diabetes at present, it is susceptible to short-term lifestyle changes, such as diet and activity before the test. HbA1c is a class of stable compounds in which glucose is covalently bonded to the N-terminal valine residues of the β-chain of hemoglobin (18). It can accurately reflect the long-term blood glucose levels over the past 2–3 months and is not easily affected by short-term fluctuations in the blood glucose concentration, and thus it is used as an indicator in the diagnosis and monitoring of diabetes (19). In addition, blood can be collected at any time to measure HbA1c levels. This is typically a non-fasting test and no special preparation is needed, and the diagnosis can be made on the day of treatment (20). Therefore, the role of HbA1c in the management of gestational diabetes has received increasing attention. It is not only a core indicator for the evaluation of hypoglycemic efficacy, but is also an indispensable indicator in the development of clinical trials related to gestational diabetes-related pregnancy outcomes (21). Studies have shown that elevated first- or second-trimester HbA1c levels are associated with adverse pregnancy outcomes (21, 22). The Classification of Diabetes Mellitus issued by the World Health Organization in 2019 recommends the use of HbA1c to diagnose diabetes in countries and regions where equipment is available, and the HbA1c cut-off point for the identification of diabetes is ≥6.5%. According to the ADA's 2016 Standards of Care, under ideal circumstances, the HbA1c value of <6% (42 mmol/mol) is optimal during pregnancy, if it can be achieved without significant hypoglycemia (23). Therefore, we set the cut-off value of 6% for HbA1c grouping in this study based on the above-mentioned criteria. Based on this cut-off value, our study found that pregnant women with GDM in the elevated HbA1c group in the second trimester of pregnancy had a significantly higher risk of gestational hypertension, postpartum hemorrhage, placental abruption, neonatal asphyxia, and macrosomia when compared to those of the normal HbA1c group.

In pace with the improvement of living standards and attaching importance to nutrition during pregnancy, recently, researchers have been paying more attention to the appropriate GWG in perinatal health care. For instance, the 2009 IOM revised guidelines for gestational weight gain provide clinicians with a practical basis. The guidelines recommend an optimal amount of GWG for pregnant women according to pre-pregnancy BMI (underweight, normal weight, overweight, and obese) (24). Although some domestic scholars have taken the differences between Chinese and western women in terms of body shape, dietary structure, cultural beliefs, and practices of pregnancy into account and thereby formulated the range of GWG according to the classification of Chinese adult BMI (25), Rebecca F Goldstein et al. (26) conducted a meta-analysis of more than one million pregnant women with gestational diabetes and found that the IOM guidelines were applicable to the pregnant women of the United States, Western Europe, and East Asia. Therefore, GWG was still categorized according to the IOM recommendations in this study. Several studies have previously shown that GWG is associated with gestational diabetes. Qi et al. (27) analyzed 8,356 pregnant women and found that excessive weight gain in the first and second trimester of pregnancy is a high-risk factor for gestational diabetes. Moreover, a large number of studies have shown that excessive GWG is a risk factor for various adverse maternal and infant outcomes in pregnant women with diabetes (28, 29). Research by Kim et al. (30) demonstrated that excessive weight gain during pregnancy is an independent predictor of the occurrence of fetal macrosomia, while appropriate GWG reduces the occurrence of fetal macrosomia in pregnant women with gestational diabetes by one-third. Xueqin Zhang et al. (31) pointed out that excessive weight gain in the second trimester of pregnancy is a high-risk factor for large-for-gestational-age (LGA) infants, whereas excessive weight gain in the third trimester of pregnancy is a high-risk factor for preeclampsia and cesarean section. A systematic review conducted by Rebecca F Goldstein et al. (29) reported that inadequate GWG is a high-risk factor for small-for-gestational-age (SGA) infants. Our study showed that excessive GWG is a high-risk factor for primary cesarean section, hypertensive disorders during pregnancy, and macrosomia, which is consistent with the previous research reports. In view of this, it is apparent that appropriate weight management during pregnancy is also crucial for improving perinatal outcomes.

In general, weight management during pregnancy plays an important role in reducing adverse pregnancy outcomes. However, a few studies were conducted at home and abroad to determine whether weight management during pregnancy can still work for gestational diabetic women with elevated HbA1c levels. Lise L. Kurtzhals et al. (32) revealed that the pregnant women with GDM who gained excessive weight had a significant increase in HbA1c in the third trimester and a significant increase in newborn birth weight, compared to patients with well-controlled weight gain. In pregnant women with strict weight control, HbA1c in the third trimester of pregnancy was significantly reduced without increasing the incidence of SGA infants. Combined with prior results, we speculated that GWG and HbA1c levels might have an interactive effect on the pregnancy outcomes in pregnant women with gestational diabetes. Therefore, we analyzed the influence of HbA1c on pregnancy outcomes in each group of pregnant women with stratification of GWG. The results showed that when the GWG was inadequate or appropriate, there was no significant difference in the risk of postpartum hemorrhage, placental abruption, neonatal asphyxia, and macrosomia between the two groups of pregnant women with GDM. However, among pregnant women with excessive GWG, the risk of postpartum hemorrhage, neonatal asphyxia, and macrosomia in the elevated HbA1c group was significantly increased. In addition, even if the GWG was appropriate, the risk of hypertensive disorders during pregnancy in the elevated HbA1c group was still higher than that observed in the normal HbA1c group. Therefore, it is confirmed that appropriate weight management during pregnancy is essential. Moreover, monitoring and controlling of the blood glucose levels may reduce the risk of adverse pregnancy outcomes among those GDM women who had excessive GWG. We recommend that HbA1c should also be considered as an important indicator of glycemic control for GDM women, particularly for those who gained excessive weight during pregnancy, to further reduce the occurrence of adverse pregnancy outcomes.

However, our study has some limitations. First, given that it is a retrospective study, selection and recall bias was inevitable. Second, the population included in this study was pregnant women attending the Women's Hospital School of Medicine, Zhejiang University, and the sample suggests a lack of representativeness. In addition, the sample size was inadequate, as there were only 49 pregnant women with GDM in the elevated HbA1c group. Moreover, the lack of data on HbA1c levels in the third trimester of pregnancy made it impossible to study the effect of gestational weight gain on this parameter. The above-mentioned deficiencies need to be addressed in further follow-up research.

In summary, the second-trimester HbA1c level is an important indicator for predicting adverse pregnancy outcomes in pregnant women with gestational diabetes, and therefore should be taken into account in the regular obstetric examination. Meanwhile, weight management during pregnancy significantly improves pregnancy outcomes, and reasonable weight gain suggestions should be given to the pregnant women in the first trimester of pregnancy based on pre-pregnancy BMI. For pregnant women with GDM who gained excessive weight during pregnancy, it is particularly necessary to conduct obstetric examination and follow-up education, monitor and control blood glucose levels, and strictly manage the weight during pregnancy, in order to further reduce the occurrence of adverse pregnancy outcomes.

## Data Availability Statement

The raw data supporting the conclusions of this article will be made available by the authors, without undue reservation.

## Ethics Statement

The studies involving human participants were reviewed and approved by Human Ethics Committee at Women's Hospital, School of Medicine, Zhejiang University. The patients/participants provided their written informed consent to participate in this study.

## Author Contributions

Conceptualization of the study is done by QZ and CL. Data curation is performed by QZ and LZ. Formal analysis and investigated the study are made by QZ, CL, LZ, and QW. QZ contributed to funding acquisition, project administration, and methodology. CL and QW contributed to resources. YC, DC, and LQ supervised the study. QZ, CL, LZ, YC, DC, and LQ validated the study and wrote the original draft. CL performed visualization. QZ, CL, and ZL contributed to writing, reviewing, and editing the manuscript. All authors contributed to the article and approved the submitted version.

## Funding

This work was supported by grants from the National Natural Science Foundation of China (81974234).

## Conflict of Interest

The authors declare that the research was conducted in the absence of any commercial or financial relationships that could be construed as a potential conflict of interest.

## Publisher's Note

All claims expressed in this article are solely those of the authors and do not necessarily represent those of their affiliated organizations, or those of the publisher, the editors and the reviewers. Any product that may be evaluated in this article, or claim that may be made by its manufacturer, is not guaranteed or endorsed by the publisher.
